# SPNS1 ablation drives skeletal muscle atrophy by disrupting mitophagy, mitochondrial function, and apoptosis in mice

**DOI:** 10.1016/j.gendis.2023.101083

**Published:** 2023-09-07

**Authors:** Xianzhong Zhang, Fengmin Zhang, Haofan Wu, Zhen Yu, Chengle Zhuang

**Affiliations:** a Department of Gastrointestinal Surgery, Shanghai Tenth People's Hospital, School of Medicine, Tongji University, Shanghai 200092, China; b Colorectal Cancer Center, Shanghai Tenth People's Hospital, School of Medicine, Tongji University, Shanghai 200072, China

Sarcopenia is characterized by a dramatic decline in muscle mass and function during aging, which often leads to reduced mobility, diminished quality of life, and shortened survival. Despite recent advances in sarcopenia, the mechanisms that mediate skeletal muscle dysfunction remain unclear.^1^ Lysosomal plays a vital role in digesting and recycling macromolecules, and several studies have shown that lysosomal acidification and autophagy decrease with age, which may impair the clearance of damaged mitochondria.^2^ SPNS1 (spinster homolog 1), a proton-dependent lysophosphatidylcholine and lysophosphatidylethanolamine transporter, plays a critical role in maintaining normal lysosomal function.^3^ However, the physiological relevance of lysosomal dysfunction in sarcopenia remains unclear. Coffey et al found that loss of SPNS1 dysregulated lysosomal pH, which impacts the expression of extracellular matrix proteins at the myotendinous junction in zebrafish.^4^ In this study, we demonstrate that ablation of SPNS1 in skeletal muscle results in impaired lysosomal digestion, hindering mitophagy and triggering the accumulation of abnormal mitochondria. As a result, damaged mitochondria cannot be cleared, leading to the leakage of pro-apoptotic protein and the activation of apoptosis. Our findings reveal the role of SPNS1 in maintaining mitochondrial-lysosomal homeostasis and preserving muscle mass and function.

Initially, we assessed the expression levels of SPNS1 in the gastrocnemius of young (16 weeks old) and middle-aged (53 weeks old) wild-type C57BL/J mice. The results revealed a decreasing trend in the protein expression of SPNS1 in the older mice (Fig. S1A, B). As age-related reductions in muscle mass and mitophagy preferentially occur in type II muscle fiber,^3^ we generated a conditional knockout mouse model of *SPNS1* gene in type II muscle fibers (*Myl1-Cre*; *SPNS1*^*flox/flox*^) to explore the associations between lysosomal and mitochondrial dysfunction and the development and progression of sarcopenia. The construction and identification of SPNS1 conditional knockout in type II muscle fibers are displayed in Figure S1C–E. Middle-aged (53 weeks) *SPNS1*^*−/−*^ mice showed significantly lower body size, weight, and daily food consumption than age-matched SPNS1^f/f^ mice (Fig. S1F, G). Additionally, *SPNS1*^*−/−*^ mice had more widespread white hairs and more severe kyphosis compared with age-matched *SPNS1*^*f/f*^ mice (Fig. 1A). Physical assessments showed that *SPNS1*^*−/−*^ mice exhibited significantly lower physical performance (Fig. S1H–K). These results indicate that the knockout of SPNS1 in type II muscle fibers accelerates skeletal muscle function decline and aging-related phenotypes.

**Figure 1:**
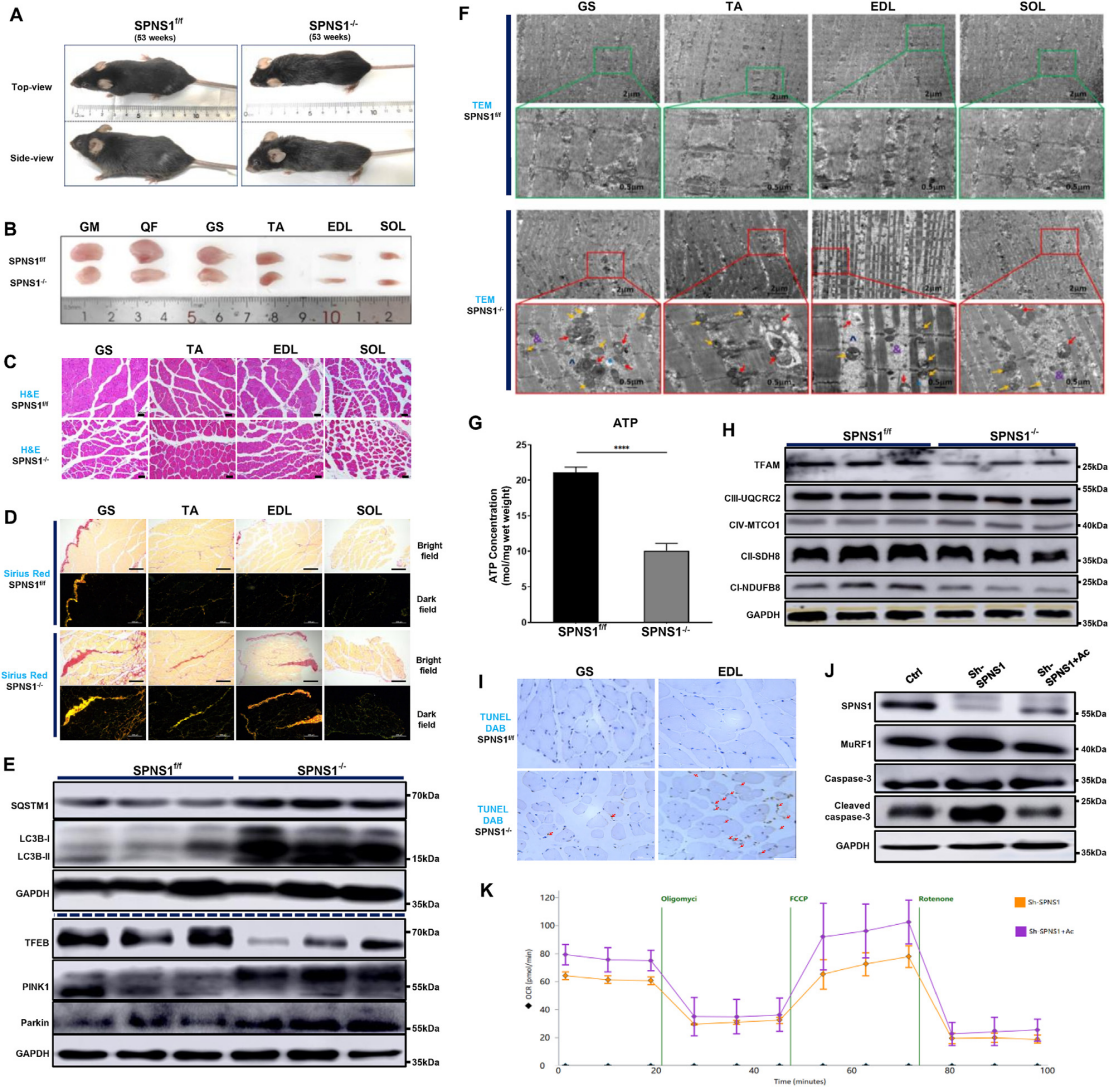
SPNS1 ablation drives skeletal muscle atrophy by disrupting mitophagy, mitochondrial function, and apoptosis in mice. **(A)** Representative images of 53-week-old *SPNS1*^f/f^ and *SPNS1*^−/−^ mice. White hairs were more widespread and kyphosis was more severe in *SPNS1*^*−/−*^ mice. **(B)** Representative images of GM, QF, GS, TA, EDL, and SOL. Muscle mass in GM, QF, GS, TA, and EDL were decreased in *SPNS1*^*−/−*^ mice. The mass of SOL showed no significant difference between the two groups. **(C)** H&E staining of GS, TA, EDL, and SOL tissue sections. The muscle fiber diameter of GS, TA, and EDL was significantly decreased in *SPNS1*^*−/−*^ mice, but SOL was unaltered. **(D)** Bright-field and dark-field imaging of Sirius red staining. Deletion of SPNS1 in muscle increased type I and type III collagen in GS, TA, and EDL, but no change was observed in SOL. **(E)** Western blotting of SQSTM1/p62, LC3B, TFEB, PINK1, and Parkin of mouse EDL protein extracts, with the protein expression normalized using GAPDH as a loading control. The content of LC3B-II, SQSTM/p62, and LC3B–I/LC3B-II ratio were higher, and the content of TFEB was lower in *SPNS1*^*−/−*^ mice. The protein levels of PINK1 and Parkin were significantly elevated in the *SPNS1*^*−/−*^ group. **(F)** Representative transmission electron micrographs of GS, TA, EDL, and SOL. SPNS1 deletion in muscle revealed a reduction of myofibril diameter and accumulation of glycogen granules, lipid droplets, and enlarged autolysosome. Remarkable heterogeneity was also observed in mitochondrial structure, with many disturbed mitochondrial ultrastructures showing abnormal cristae organization, matrix dissolution, degeneration, and vacuolization. &, broken muscle fibers; yellow arrow, abnormal mitochondria; red arrow, abnormal autopolysosome; ∗, lipid droplet; ˆ, electron-dense material. **(G)** Adenosine triphosphate (ATP) concentration assay of mouse EDL protein extracts. ATP production was significantly reduced in *SPNS1*^*−/−*^ group (*n* = 6). **(H)** Western blotting of mitochondrial transcription factor A (TFAM) and mitochondrial respiratory complex I, complex II, complex III, and complex IV of mouse EDL protein extracts, with the protein expression normalized using GAPDH as a loading control. The protein content of TFAM, complex I, and complex II were significantly down-regulated in the *SPNS1*^*−/−*^ group. **(I)** TUNEL staining of mouse EDL. Loss of SPNS1 in muscle increased the proportion of TUNEL^+^ apoptotic muscle fibers (red arrow). **(J)** Western blotting of SPNS1, MuRF1, caspase-3, and cleaved caspase-3 in C2C12 myotubes in different groups. Knockdown of SPNS1 in C2C12 myotubes significantly increased the protein level of MuRF1 and cleaved caspase-3, which was rescued by Ac-DEVD-CHO. **(K)** Oxygen consumption rate (OCR) of C2C12 myotubes in different groups. Loss of SPNS1 significantly reduced basal respiration and maximal respiration in C2C12 myotubes, which was partly rescued by Ac-DEVD-CHO.

Subsequently, the mice were sacrificed, and the muscles in their hind limb were isolated according to muscle groups. *SPNS1*^*−/−*^ mice had smaller muscle mass in the gluteus maximus (GM), quadriceps (QF), gastrocnemius (GS), tibialis anterior (TA), and extensor digitorum longus (EDL) compared with SPNS1^f/f^ mice. The mass of the soleus (SOL), which is mainly composed of slow-type fibers, showed no significant difference between the two groups (Fig. 1B; Fig. S2A). The above conclusion was also confirmed by analyzing muscle hematoxylin and eosin staining and quantifying the muscle fibers' cross-sectional area (Fig. 1C; Fig. S2B). Consistently, Western blotting analysis of EDL in the *SPNS1*^*−/−*^ groups demonstrated an increased protein content of catabolism (Ubiquitinylated proteins, Atrogin-1and MuRF1), and slow-type I fibers (Myh7), decreased protein content of anabolism (p70S6K and 4E-BP1), and fast-type IIa fibers (Myh2) (Fig. S2C, D). Furthermore, the results of Masson and Sirius red staining and Western blotting analysis revealed increased fibrous tissue with a higher type I and type III collagen volume fraction in GS, TA, and EDL in *SPNS1*^*−/−*^ mice (Fig. 1D; Fig. S3A–C).

Next, we investigated the effects of SPNS1 deletion on lysosomal function. Western blotting results of EDL showed that the content of LC3B-II, SQSTM/p62, and LC3B–I/LC3B-II ratio were higher, and the content of transcription factor EB (TFEB) was lower in *SPNS1*^*−/−*^ mice (Fig. 1E; Fig. S4A), indicting normal autophagy initiation but impaired lysosomal degradation and biogenesis function. In addition, we also examined the levels of lysosomal proteases, cathepsin B, and cathepsin D. The results showed a decreasing trend in cathepsin B levels in *SPNS1*^*−/−*^ mice, while no significant changes were observed in cathepsin D levels (Fig. S4B, C). Mitophagy is important for regulating mitochondrial number and maintaining mitochondrial function, and normal lysosomal function plays a vital role in the removal of damaged mitochondria. We then tested the expression of mitophagy-related proteins and found that the protein levels of PINK1 and Parkin were also significantly elevated in the *SPNS1*^*−/−*^ group, indicating dysfunction of mitophagy and accumulation of damaged mitochondria (Fig. 1E; Fig. S4A).

We also assessed the ultrastructure changes of GS, TA, EDL, and SOL by transmission electron microscopy. The analysis of the muscles from *SPNS1*^*−/−*^ mice revealed a reduction in myofibril diameter and the accumulation of glycogen granules, lipid droplets, and enlarged autolysosome. We speculate that the increased number of lipid droplets in *SPNS1*^*−/−*^ muscle samples may be related to the lipid transport function of SPNS1. More importantly, remarkable heterogeneity was observed in mitochondrial structure with much disturbed mitochondrial ultrastructure, which showed abnormal cristae organization, matrix dissolution, degeneration, and vacuolization (Fig. 1F; Fig. S5A). At the tissue level, the indirect measurement of mitochondrial function by detecting the total amount of adenosine triphosphate showed a significant decrease in adenosine triphosphate levels in EDL of *SPNS1*^*−/−*^ mice (Fig. 1G), along with the down-regulation of mitochondrial transcription factor A, respiratory chain complex I and II (Fig. 1H; Fig. S5B). All these findings demonstrated that lysosomal dysfunction due to SPNS1 knockout could impede mitophagy and disrupt mitochondria function. As the main organelles involved in nutrient metabolism and energy homeostasis, lysosomes and mitochondria are rich sources of reactive oxygen species. We then examined intracellular oxidative stress levels of the two groups and found that the content of malondialdehyde, a biomarker of oxidative stress, was increased, and major endogenous antioxidative molecule Nrf2 and its downstream targeted proteins SOD1 and HO-1 were significantly decreased in the EDL of *SPNS1*^*−/−*^ group by Western blotting analysis (Figs. S5C and D). The decline of antioxidant capacity in EDL of *SPNS1*^*−/−*^ mice was also determined by Trolox equivalent antioxidant capacity (Fig. S5E).

As mitochondria play a central role in apoptotic cell death, the permeable outer membrane of damaged mitochondria releases cytochrome C, which induces caspase activation and executes apoptotic cell death.^5^ We further demonstrated that *SPNS1*^*−/−*^ mice had significantly decreased anti-apoptotic BCL-2 protein expression and elevated pro-apoptotic mitochondrial protein expression, including cytochrome C and cleaved caspase-3 (Fig. S6A). TUNEL staining confirmed our results that *SPNS1*^*−/−*^ mice had an increased proportion of apoptotic muscle fibers (Fig. 1I; Fig. S6B).

To further confirm the result that SPNS1 deletion accelerates mitochondrial dysfunction and apoptosis, we knocked down SPNS1 in C2C12 cells with shRNA *in vitro*. As expected, inhibition of SPNS1 significantly increased the number of apoptotic C2C12 myoblast (Fig. S7A) and increased the protein level of MuRF1 and cleaved caspase-3 in C2C12 myotubes. The addition of an apoptosis inhibitor Ac-DEVD-CHO partly reversed the apoptosis and protein catabolism (Fig. 1J; Fig. S7B). We also assessed the capacity of oxidative phosphorylation in C2C12 myotubes with or without SPNS1 knockdown by measuring the oxygen consumption rate. We found that loss of SPNS1 significantly reduced basal respiration and maximal respiration in C2C12 myotubes, which was partly rescued by Ac-DEVD-CHO (Fig. 1K; Fig. S7C). These results suggest that lysosomal dysfunction mediated by SPNS1 loss hinders mitochondria function and leads to apoptosis.

In conclusion, our findings indicate that the conditional knockout of SPNS1 in type II muscle exacerbates the aging phenotype and leads to muscle fiber atrophy and significant skeletal muscle fibrosis. Loss of SPNS1 impairs the autophagy-lysosomal system, as evidenced by the accumulation of LC3B-II, p62, swollen lysosome, and impaired mitochondria. Our data suggest a defect in mitophagy, which leads to the accumulation of damaged mitochondria and subsequent release of pro-apoptotic proteins, ultimately activating apoptosis. The specific mechanism by which SPNS1 deficiency leads to mitophagy and lysosomal dysfunction requires further investigation. Our study successfully established a mouse model for investigating the mechanisms of lysosome- and mitochondrion-related muscle diseases, such as sarcopenia and muscular lysosomal storage disorders.

## Ethics declaration

All animal procedures were conducted in accordance with institutional guidelines approved by the Institutional Animal Committee of Tongji University. The mice used in this study were cared for by the Guide for the Care and Use of Laboratory Animals throughout the experiment.

## Author contributions

Conceptualization: C.Z. Formal analysis: X.Z., F.Z., and H.W.; Funding acquisition: C.Z.; Investigation: X.Z., F.Z., and H.W.; Methodology: X.Z. and F.Z.; Project administration: Z.Y. and C.Z.; Supervision: Z.Y. and C.Z.; Validation: X.Z., F.Z., and H.W.; Visualization: X.Z. and F.Z.; Writing-original draft: X.Z. and F.Z.; Writing-review & editing: X.Z., F.Z., Z.Y., and C.Z.

## Conflict of interests

The authors declare no conflict of interests.

## Funding

This work was supported by the 10.13039/501100001809National Natural Science Foundation of China (No. 82171565) and the Shanghai Chongming Science and Technology Commission of China (No. CKY2021-30).
