# Supplementary material for: SPNS1 ablation drives skeletal muscle atrophy by disrupting mitophagy, mitochondrial function, and apoptosis in mice
Source: Genes Dis. 2023 Sep 7;11(5):101083. doi: 10.1016/j.gendis.2023.101083 (PMC11145190; doi:10.1016/j.gendis.2023.101083)
Supplement: Multimedia component 1 [file mmc1.docx]

**Supplementary Data**

1. **Material and methods**

**Animals**

Eight-week-old SPNS1 muscle-specific conditional knockout (Myl1-Cre;SPNS1^-/-^, SPNS1^-/-^) mice and their counterpart control (Myl1-Cre;SPNS1^flox/flox^, SPNS1^f/f^) mice with the background of C57/BL6J were donated by Prof. Andong Qiu, School of Life Science and Technology, Tongji University, Shanghai, China. All mice were housed at 22℃ under a 12-h light-dark cycle with adequate food and water. The mice were randomized into four groups (3-4 mice per group): SPNS^f/f^ young group, SPNS^f/f^ middle-old group, SPNS1^-/-^ young group, and SPNS1^-/-^ middle-old group. All mice were injected with tamoxifen at the age of 12 weeks. The young-group and middle-old-group mice were raised until they were 16 and 53 weeks old, respectively. Protein expression of SPNS1 and muscle function was assessed in 16-week young group, and all histology and molecular biology experiments were conducted in 53-week middle-aged group. All animal procedures were conducted in accordance with institutional guidelines approved by the Institutional Animal Committee of Tongji University. The mice used for this study received care throughout the experiment following the Guide for the Care and Use of Laboratory Animals.

**Cell culture**

C2C12 mouse myoblasts purchased from ATCC (Manassas, VA, USA) were cultured in growth media (Dulbecco’s modified Eagle’s medium (DMEM) with 10% fetal bovine serum (FBS) and 1% penicillin-streptomycin (PS) under a humidified atmosphere at 37℃ in an incubator. Knockdown of SPNS1 in C2C12 myoblasts were performed by shRNA at 24h-48h after seeding, along with the addition of the caspase-3 inhibitor Ac-DEVD-CHO (10 μM, MedChemExpress) when needed. When cells reached 70% confluency, C2C12 myoblasts were cultured with DMEM differentiation medium containing 2% horse serum and 1% PS to differentiate into myotubes for either 3 or 6 days.

**Assessment of skeletal muscle function in mice**

Grip strength test

Maximum grip strength was measured using the grip strength meter (ZhongShi Biological Technology Co. Ltd., China). Mice were placed on the grip strength meter with all four paws on the grid. During the test, mice were held horizontally and were slowly dragged backwards until they lost their grip. Five trials were performed at 1-minute intervals, with the average value after excluding maximum and minimum values as the grip strength.

Treadmill test

Mice were acclimated to a treadmill device (SANS Biological Technology Co. Ltd., China) with a slope of 10% for two consecutive days before the test. The treadmill endurance test consisted of a warm-up exercise of 5m/min for 6 minutes and a subsequent acceleration pattern of 1m/min increments until each mouse was exhausted. The test ended if the mouse received a total of 20 shocks or remained in the shock grid area for more than 2 seconds after continuous shocks. The maximum running distance and maximum running speed at fatigue were recorded.

Rotarod test

The mice were placed on the rotarod instrument (ZhongShi Biological Technology Co. Ltd., China). Once they were acclimated, an acceleration protocol (4 to 50 rpm in 300 s with directional rotation) was performed. The maximum rate of the rotarod was recorded when mice fell from the rod.

Hanging test

Mice were placed on an elevated inverted grid 50 cm above the soft bedding. Mice were placed on the grid for a while before the test to let each mouse to familiarize himself with the grid and keep their four paws griping on the grid. The time until each mouse fell off the inverted grid was recorded.

**Muscle samples collection and storage**

After physical function assessment, mice were rested for at least 3 days. Mice were weighed and anesthetized by intraperitoneal injection of 2% sodium pentobarbital (80 mg/kg), and sacrificed for sample collection. Limb muscles, including gluteus maximus (GM), quadriceps (QF), gastrocnemius (GS), tibialis anterior (TA), extensor digitorum longus (EDL), and soleus (SOL), were rapidly dissected. Specimens were frozen in liquid nitrogen, stored at -80℃, or fixed 2.5% glutaraldehyde or 4% paraformaldehyde, dehydrated in a gradient ethanol series, and embedded in paraffin to prepare 5 μm sections.

**Haematoxylin-eosin (H&E) staining**

Muscle specimens were fixed with 4% paraformaldehyde, embedded in paraffin to prepare 5μm sections, and then dewaxed and hydrated with dewaxing solution and gradient ethanol series. The specimens were soaked in hematoxylin for 3 min, then differentiated with 1% ethanol hydrochloride for 5s to make the nucleus blue, and finally soaked in eosin for 2 min to make the cytoplasm red. After dehydration with ethanol and clearing in a dewaxing solution, the slides were mounted. Images were acquired using a light microscope system (Leica, Germany). Myofibril cross-sectional area (CSA) was calculated by quantification at 100 fibers per mouse using ImageJ software.

**Transmission electron microscopy (TEM)**

Muscle samples were dissected into small bundles and fixed in 2.5% glutaraldehyde for 12h at 4℃ for 1h in 1% osmium tetroxide and dehydrated in gradient ethanol. Samples were stained with 1% uranyl lactate for 2h and dehydrated in an acetone dilution series, the samples were embedded in epoxy resin and dissected into ultrathin sections (80nm). The sections were then viewed with a transmission electron microscope (Hitachi, Japan) at 80kV. The number of mitochondria was measured in three randomly selected regions. normal mitochondria were considered if they had enlarged mitochondria or deformed mitochondrial cristae, membrane disruption, vacuolation, and matrix dissolution.

**Masson staining**

After dewaxing and hydration, the slides were soaked in ponceau magenta for 10min, 0.2% glacial acetic acid for 1 min, phosphomolybdic acid for 1 min, and finally in 0.2% glacial acetic acid for 1 min to stain the cytoplasm red. The glass slides were first treated with aniline blue for half a minute, and then soaked with 0.2% acetic acid for 1 min to dye the fibrous tissue blue. The slides were subsequently dehydrated using ethanol, cleared in a dewaxing solution, and mounted.

**Sirius red staining**

Slides were dewaxed and hydrated, then soaked in Sirius red dye solution for 1h and washed for 30s. The slides were then dehydrated using ethanol, cleared in a dewaxing solution, and mounted.

**Western blotting**

Protein was extracted from muscles, and its concentration was determined by BCA assay. Equal amounts of protein were separated by electrophoresis on a 10-12% sodium dodecyl sulfate polyacrylamide gel (SDS/PAGE). Proteins on the gels were then transferred to nitrocellulose filter membrane (NC) membranes (Millipore 66485, USA), blocked with 5% nonfat milk in Tris-buffered saline with Tween-20 (TBST) (at room temperature, 60 min), and subsequently incubated with primary antibodies [rabbit anti-SPNS1 (Novus Biologicals, NBP1-59999), rabbit anti-Atrogin 1 (Affinity Biosciences, DF7075), rabbit anti-MuRF1 (Abcam, ab172479), mouse anti-p70s6k (SANTA CRUZ, sc-8418), mouse anti- 4EBP1 (SANTA CRUZ, sc-9977), mouse anti-ubiquitin (SANTA CRUZ, sc-8017), mouse anti-MYH2 (SANTA CRUZ, sc-53095), mouse anti-MYH7 (SANTA CRUZ, sc-53089), mouse anti-Collagen Type I (Proteintech, 66761-1-Ig), mouse anti-Collagen Type III (Proteintech, 22734-1-AP), rabbit anti-TFEB (Bethyl Laboratories, A303-673A), rabbit anti-PINIK (Cell Signaling Technology, 6946), mouse anti-Parkin (Cell Signaling Technology, 4211), mouse anti-SQSTM1 (SANTA CRUZ, sc-48402), rabbit anti-LC3B (Novus Biologicals, NB100-2220), rabbit anti-Nrf2 (Abcam, ab92946), mouse anti-SOD1 (SANTA CRUZ, sc-101523), mouse anti-HO-1 (SANTA CRUZ, sc-136960), rabbit anti-MDA (Abcam, ab27642), rabbit anti- TFAM (Abcam, ab131607), mouse anti-OXPHOS (Abcam, ab110411), rabbit anti-Bcl2 (Abcam, ab182858), rabbit anti-Tom20 (Cell Signaling Technology, 42406), rabbit anti-cytochrome C (Cell Signaling Technology, 4280), rabbit anti-Casepase-3 (Cell Signaling Technology, 9662), mouse anti-Casepase-9 (Cell Signaling Technology, 9508), rabbit anti-cleaved caspase-3 (Cell Signaling Technology, 9661)]. After staining overnight at 4 °C, the membranes were washed thrice with TBST and were incubated with a secondary antibody conjugated with horseradish peroxidase (HRP) for 90 minutes at room temperature. After three more washes with TBST, enhanced chemiluminescence reagent (ECL) (Thermo Fisher Scientific A38555) was added on the membranes which was detected by ECL Western blotting detection system. Protein bands were quantified using ImageJ software.

**Oxygen consumption rate (OCR) assay**

The XF24 extracellular flux analyzer (Agilent Technologies, CA, USA) was used to evaluate mitochondrial oxygen consumption rate (OCR) of C2C12 myotubes. In short, 2×10^4^ C2C12 myotubes per well were seeded onto the XF24 Cell Culture Microplates (Agilent Technologies, Manchester, UK), and incubated for 24 h at 37°C and 5% CO_2_. Then, the cell area coverage was also checked before test to avoid the misinterpretations. Before OCR assays, the medium was changed to Agilent Seahorse XF assay medium (XF DMEM medium with 25 mM of glucose, 2 mM of L-glutamine and 1 mM of sodium pyruvate) and then the microplates were maintained in a CO_2_-free incubator for 1 h. The OCR was measured in following 90 min with 3 min interval, which were obtained at the baseline and following injection of oligomycin (1 μM), FCCP (1 μM), and a mixture of antimycin A plus rotenone (AA/ROT, 1 μM).

**Apoptosis detection**

After being treated with SPNS1 shRNA or/and Ac-DEVD-CHO, C2C12 cells were stained with an annexin V-fluorescein isothiocyanate (FITC)/propidium iodide (PI) apoptosis assay kit according to the manufacturer’s protocol (Beyotime, Beijing, China). Flow cytometry (Becton, Dickinson, Franklin Lakes, NJ, USA) was used to detect apoptosis following fluorescein isothiocyanate (FITC) and PI staining. The FlowJo software (version 7.6.1; FlowJo LLC) was used to adjust the compensation between different channels and to visualize the results.

**Statistical analysis**

Data are presented as means and standard deviation (SD). GraphPad Prism software (Version 8.0, USA) was used for statistical analysis and image presentation. Unpaired t-test was used for comparison between two groups, and one-way ANOVA followed by Bonferroni post hoc test was used for comparison between multiple groups. P value <0.05 was considered statistically significant.

1. **Supplementary figures**



**Figure S1** Development of SPNS1 conditional knockout mice and identification of muscle function. **(A-B)** Western blotting with the anti-SPNS1 antibody was used to detect SPNS1 protein expression in the gastrocnemius of 16-week and 53-week mice. The protein expression was normalized using GAPDH as a control. Data represent mean±SEM, n=4-6 animals/group. **(C)** The recombination process and the Loxp sites used for knocking out SPNS1. **(D-E)** Western blotting with the anti-SPNS1 antibody was used to detect SPNS1 protein expression in the EDL of gastrocnemius mice of SPNS1^f/f^ and SPNS1^-/-^ mice. SPNS1 expression was significantly lower in mutant mice than in controls. The protein expression was normalized using GAPDH as control. Relative expression of SPNS1. Data represent mean±SEM, n=4-6 animals/genotype, ^***^P<0.001. **(F-G)** Changes of body weight and food consumption of 53-week SPNS1^f/f^ and SPNS1^-/-^ mice. n=3-5 animals/genotype. **(H)** Hanging time of 16-week SPNS1^-/-^ and SPNS1^f/f^ mice (n=4-6 animals/genotype); **(I)** Treadmill endurance test of 16-week SPNS1^f/f^ and SPNS1^-/-^ mice (n=4-6 animals/genotype); **(J)** Grip strength test of 16-week SPNS1^f/f^ and SPNS1^-/-^ mice (n=4-6 animals/genotype); **(K)** Rotaroad test of 16-week SPNS1^f/f^ and SPNS1^-/-^ mice (n=4-6 animals/genotype). Data represent mean±SEM, ^**^P<0.01, ^***^P<0.001, ^****^P<0.0001.


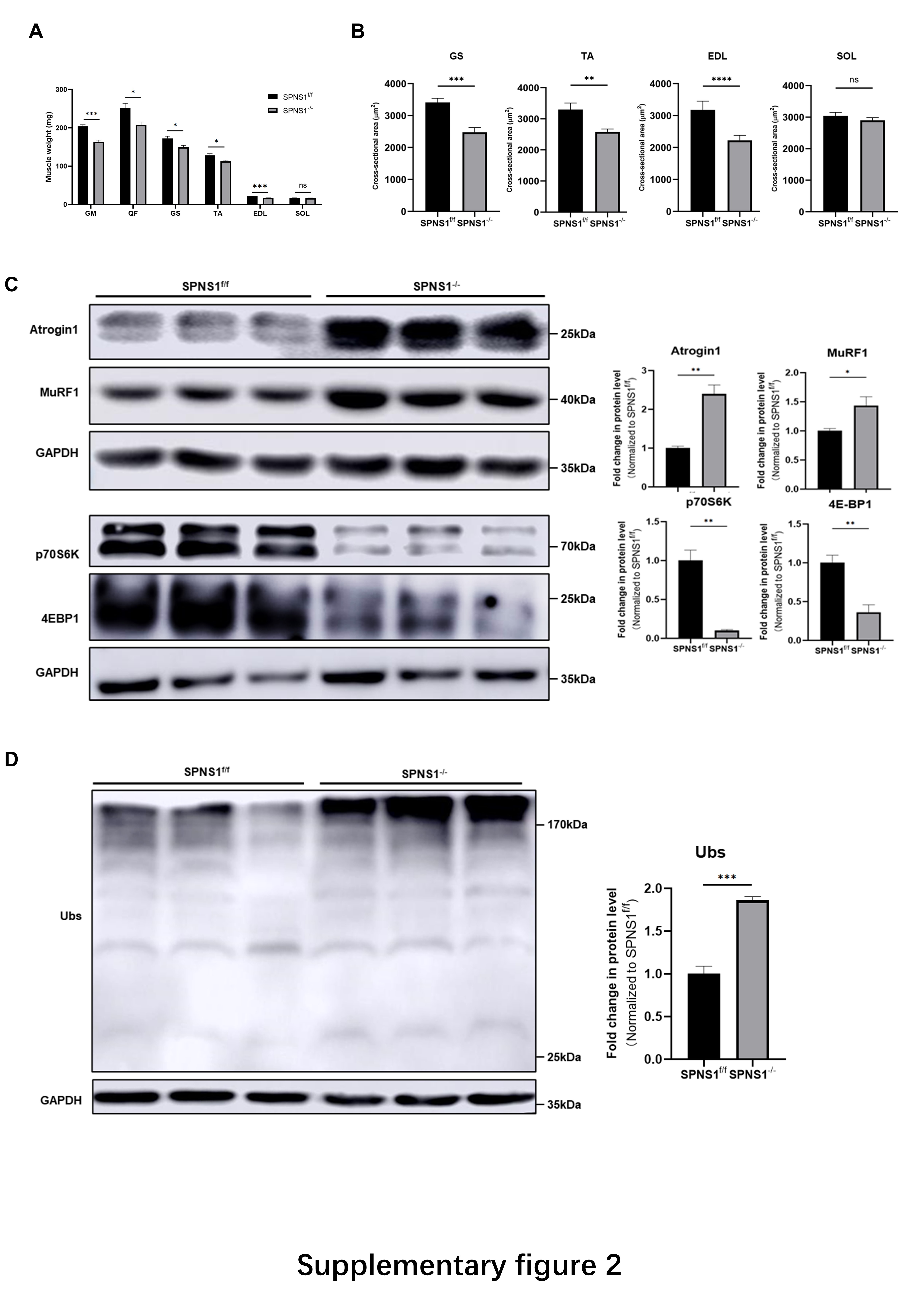


**Figure S2** Skeletal muscle‐specific knockout of SPNS1 induces skeletal muscle atrophy and proteostasis-imbalance in mice. **(A)** Statistical results of muscle weight of GS, TA, EDL and SOL. **(B)** Cross-sectional area of GS, TA, EDL and SOL muscle fibres. **(C)** Western blotting of Atrogin1, MuRF1, p70S6K and 4E-BP1 of mouse EDL protein extracts from 53-week SPNS1^f/f^ and SPNS1^-/-^ mice and relative expression of Atrogin1, MuRF1, p70S6K and 4E-BP1 was normalized using GAPDH as control. **(D)** Western blotting of ubiquitinated proteins of mouse EDL protein extracts from SPNS1^f/f^ and SPNS1^-/-^ mice and relative expression of ubiquitinated proteins was normalized using GAPDH as control. Data represent mean±SEM, n=4-6 animals/genotype, ^*^P<0.05, ^**^P<0.01, ^***^P<0.001, ^****^P<0.0001, ns not significant.


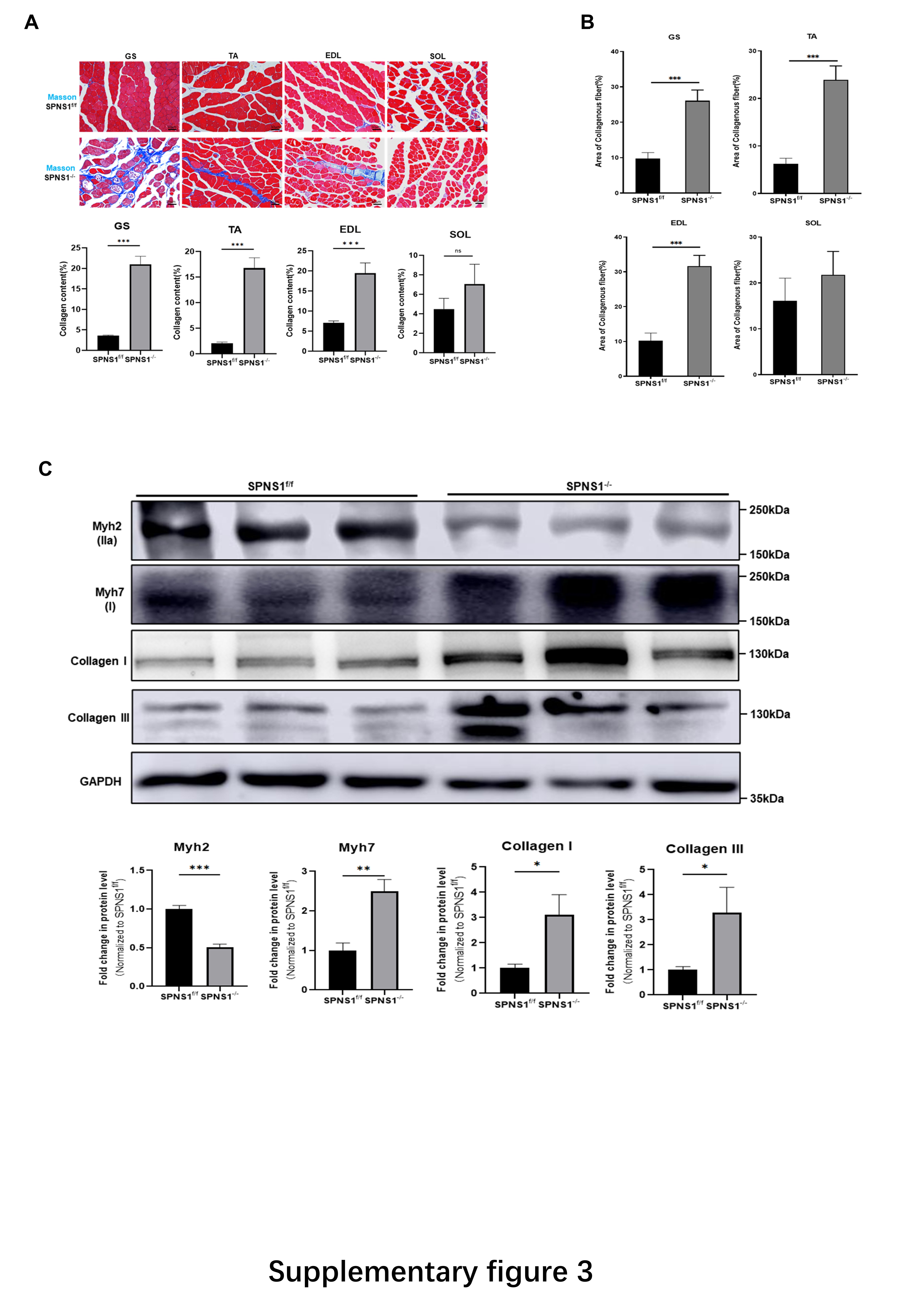
**Figure S3** Skeletal muscle‐specific knockout of SPNS1 induce collagen deposition. **(A)** Masson staining of intramuscular interstitial fibrosis and relative collagen content of GS, TA, EDL and SOL by Masson staining. **(B)** Relative collagen content of GS, TA, EDL and SOL by Sirius red staining. **(C)** Western blotting of Myh2, Myh7, Collagen I and Collagen III of EDL protein extracts from SPNS1^f/f^ and SPNS1^-/-^ mice and relative expression of Myh2, Myh7, Collagen I and Collagen III was normalized using GAPDH as control. Data represent mean±SEM, n=4-6 animals/genotype, ^*^P<0.05, ^**^P<0.01, ^***^P<0.001, ^****^P<0.0001, ns not significant.


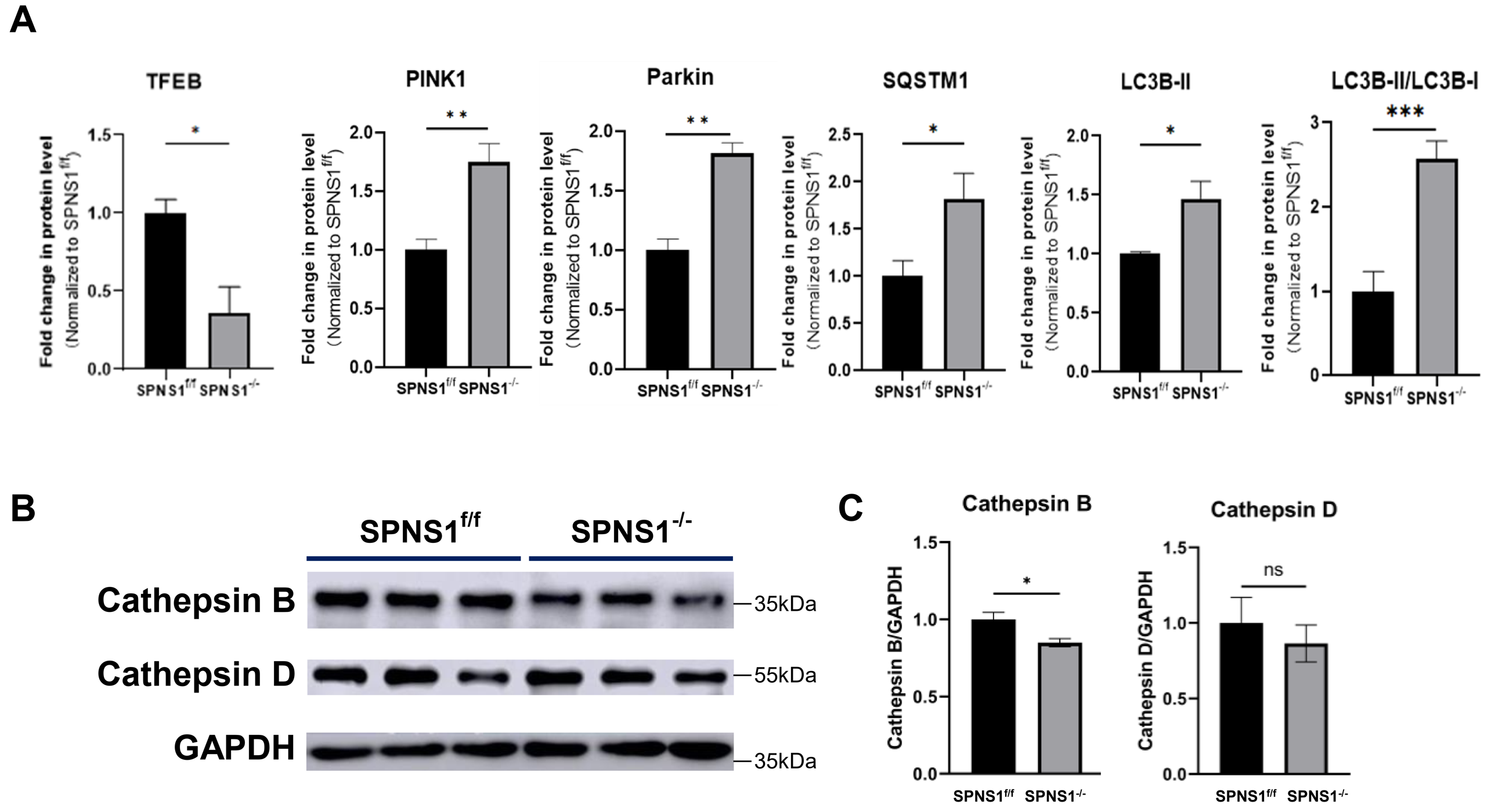


**Figure S4** Skeletal muscle‐specific knockout of SPNS1 impaired lysosomal function and autophagic degradation. **(A)** Relative expression of TFEB, PINK1, Parkin, SQSTM1/p62, LC3B-I and LC3B-II was normalized using GAPDH as control. **(B-C)** Western blotting of cathepsin B and cathepsin D of mouse EDL protein extracts from SPNS1^f/f^ and SPNS1^-/-^ mice, and relative expression of cathepsin B and cathepsin D was normalized using GAPDH as control.





**Figure S5** Skeletal muscle‐specific knockout of SPNS1 resulted in impaired mitochondrial morphology and function, leading to increased oxidative damage in mice. **(A)** Statistical results of the number of lipid droplets, autophagosomes, and the percentage of abnormal mitochondria. **(B)** Relative expression of TFAM, CI, CII, CIII and CIV was normalized using GAPDH as control. **(C-D)** Western blotting of Nrf2, SOD1, HO-1 and MDA of mouse EDL protein extracts from SPNS1^f/f^ and SPNS1^-/-^ mice, and relative expression of Nrf2, SOD1, HO-1 and MDA was normalized using GAPDH as control. **(E)** Total antioxidant capacity of mouse EDL protein extracts from SPNS1^f/f^ and SPNS1^-/-^ mice. Data represent mean±SEM, n=4-6 animals/genotype, ^*^P<0.05, ^**^P<0.01, ^***^P<0.001.


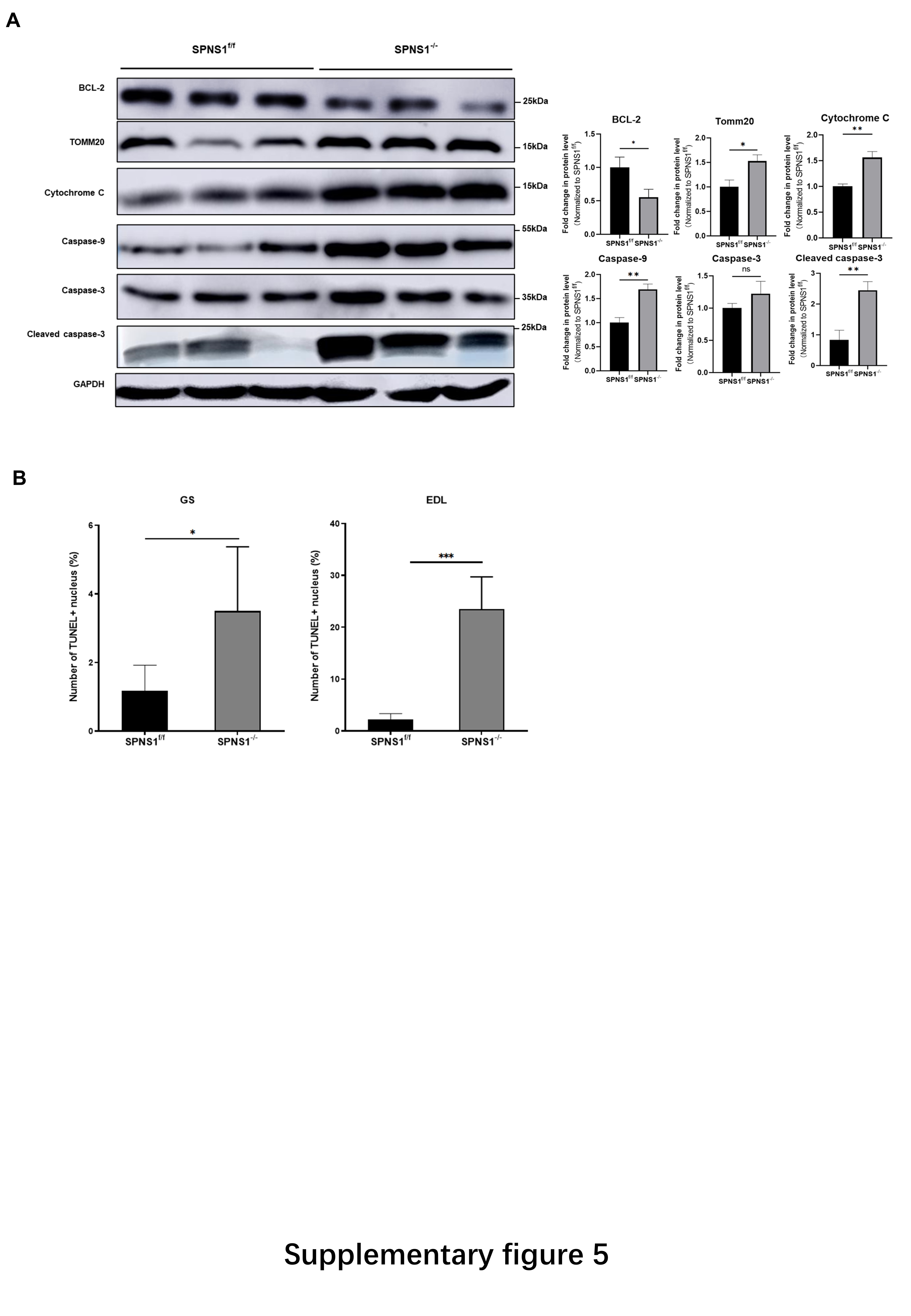
**Figure S6** Skeletal muscle‐specific knockout of SPNS1 results in mitochondrial dysfunction and activation of mitochondrial apoptosis through the caspase-3 pathway. **(A)** Western blotting of BCL-2, TOMM20, cytochrome C, caspase-9, caspase-3 and cleaved caspase-3 of mouse EDL protein extracts from SPNS1^f/f^ and SPNS1^-/-^ mice, and relative expression of BCL-2, TOMM20, cytochrome C, caspase-9, caspase-3 and cleaved caspase-3 was normalized using GAPDH as control. **(B)** Relative proportion of apoptotic TUNEL^+^ nucleus in GS and EDL. Data represent mean±SEM, n=4-6 animals/genotype, ^*^P<0.05, ^**^P<0.01, ^***^P<0.001.


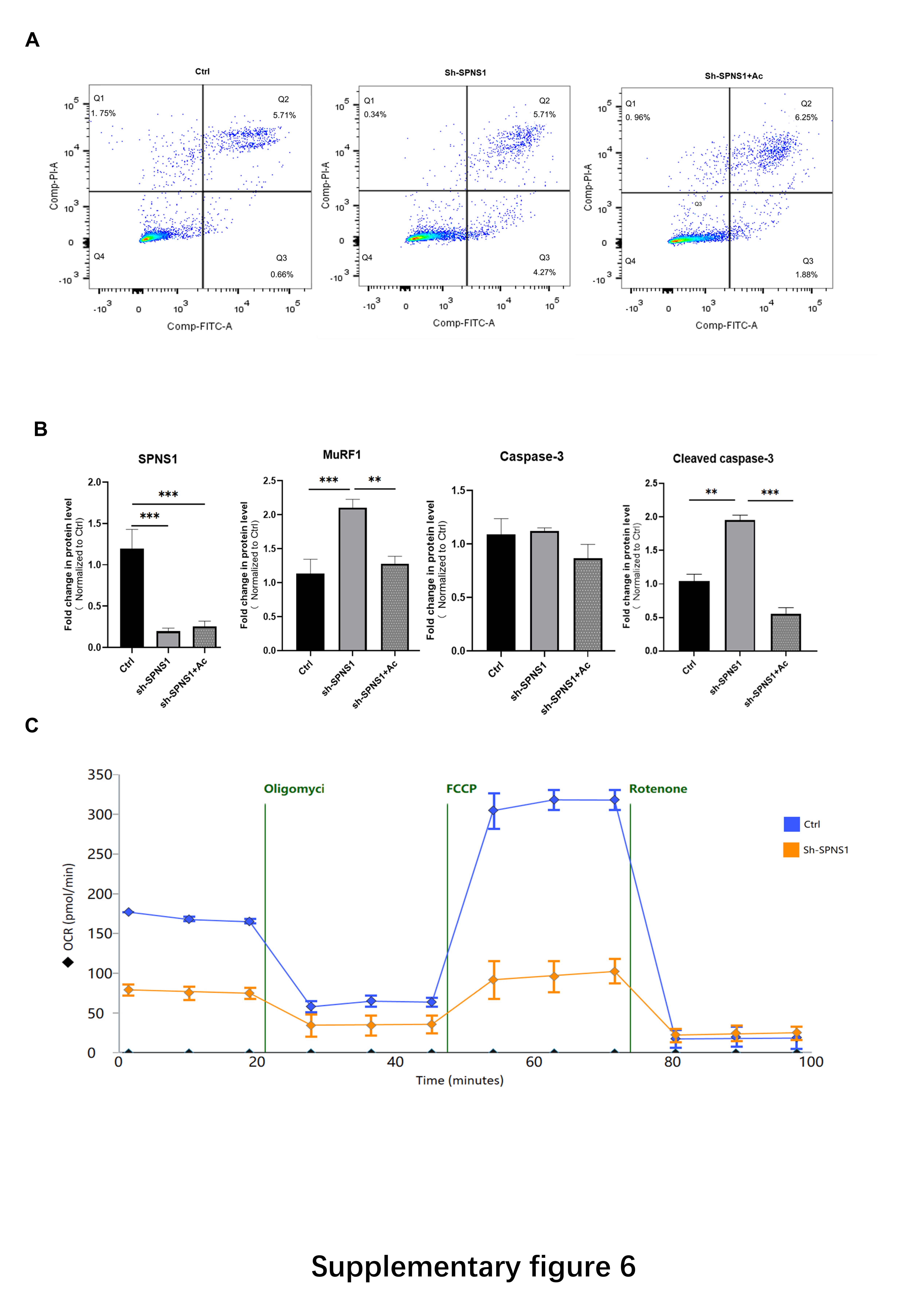
**Figure S7** Knockdown of SPNS1 increases apoptosis and impairs mitochondrial oxidative phosphorylation in C2C12 cells. **(A)** Representative flow cytometry plots and quantitative analysis of cell apoptosis. **(B)** Relative expression of SPNS1, MuRF1, caspase-3, and cleaved caspase-3 in C2C12 myotubes in different groups was normalized using GAPDH as control. **(C)** Oxygen consumption rate (OCR) of C2C12 myotubes in different groups. Data represent mean±SEM, n=4-6, ^**^P<0.01, ^***^P<0.001, ns not significant.
